# Progresses and clinical application of super-resolution ultrasound imaging: a narrative review

**DOI:** 10.1186/s13089-025-00432-6

**Published:** 2025-06-16

**Authors:** Jia-yi Gao, Chao Hou

**Affiliations:** Department of Ultrasound, the Affiliated Hospital, Southwest Medical University, Luzhou, 646000 China

**Keywords:** Super-resolution ultrasound, Microcirculation, Microbubble

## Abstract

Microcirculation plays a crucial role in maintaining normal physiological functions in the human body by facilitating the exchange of materials between tissues and blood through a network of microvessels with diameters less than 100 μm. It regulates local hemodynamics and participates in important pathophysiological processes, such as inflammatory reactions and immune responses. In recent years, the monitoring of super-resolution ultrasound (SRUS) in microcirculation has significantly enhanced our understanding of microvascular structure and function, while also providing insights into the noninvasive evaluation of organ conditions at the micro-level, thereby promoting the diagnosis and treatment of related diseases. This review summarizes the development and clinical application progress of SRUS, offering valuable insights into future research directions.

## Background

Microcirculation refers to the blood circulation that occurs between arterioles and venules. It is characterized by capillaries with a diameter of less than 10 μm, where the exchange of substances between blood and tissue cells takes place. When structural or functional impairments in microcirculation lead to a reduction in microcirculatory blood perfusion, the supply of nutrients and oxygen becomes insufficient to meet the metabolic demand of tissues. Therefore, the structural and hemodynamic conditions of the microvasculature play a crucial role in diagnosing and assessing a wide range of diseases and pathologies [1, 2]. Abnormal changes in the structure or function of the microvascular system have been identified as key evidence for the development of several critical diseases, including cancer, inflammation, atherosclerotic plaques, and neurodegenerative disorders, etc. [3]. Multimodal ultrasound technologies, including color Doppler flow imaging (CDFI), contrast-enhanced ultrasound (CEUS), and super microvascular imaging (SMI), facilitate noninvasive monitoring of circulatory information pertinent to the functional status of organ structures. However, these methods exhibit certain limitations. For instance, CDFI is restricted to visualizing vessels at the millimeter scale and is susceptible to the angle of the ultrasound beam. CEUS relies on the backscatter intensity of microbubble (MB), which may be affected by biocompatibility and the surrounding microenvironment, complicating the differentiation between noise and artifacts in practical applications [4]. Furthermore, both CEUS and SMI encounter challenges in identifying features smaller than the wavelength of ultrasound, thereby constraining their ability to visualize capillaries. In recent years, a novel ultrasound technology known as super-resolution ultrasound imaging (SRUS) has emerged, which isolates source echoes that exceed the classical diffraction limit [5]. This technology effectively tracks MB signals and their motion trajectories within blood flow, generating images that accurately reflect their positional information. Such capabilities facilitate the measurement of density and velocity distributions within the microvascular system, enabling the visualization and quantification of blood flow information at a micro level. Currently, SRUS has been applied in various animal experiments, with several instruments having been commercialized [6, 7], demonstrating the potential of using SRUS in clinical practice. Consequently, this article aims to review the latest advancements in the applications of SRUS.

## Proposal of the concept of SRUS

SRUS originated in the 1980s as a technique for separating echoes from sources located closer than the classical diffraction limit [8]. Its resolution is constrained by the wavelength; a decrease in wavelength leads to a significant increase in ultrasound absorption, which consequently limits imaging depth. Therefore, in clinical applications, the resolution limit of ultrasound imaging is typically maintained at the hundred-micron level [9]. The advancement of SRUS microvascular imaging has been facilitated by progress in optical imaging technology. In 2006, novel methods capable of overcoming the optical diffraction limit were introduced, including fluorescence photoactivated localization microscopy, photoactivated localization microscopy, and stochastic optical reconstruction microscopy [10–12]. Drawing inspiration from these developments, SRUS technology achieved spatial imaging with sub-wavelength resolution by determining the center of mass of each random scintillation fluorescence source and leveraging the systematic point spread function (PSF), in conjunction with sequential datasets captured by a high-speed camera, resulting in spatial resolution on the order of tens of nanometers. In 2009, Zheng et al. proposed a method for measuring flow velocity in small blood vessels using CEUS with harmonic ultrasound imaging to track the motion of MB at a high frame rate [13]. This technique mitigates mutual interference between MB by observing them sequentially, which allows for the identification of isolated sound sources in each frame. When the radio frequency (RF) channel data or the PSF of the beam-forming image is known, MBs can be localized with high precision. This precision enables the generation of super-resolution microvascular images by accumulating subwavelength localization information. In 2011 [14], Couture et al. proposed the concept of using ultrasound localization microscopy (ULM) as an equivalent method to photoactivated localization microscopy and they validated its localization resolution in vivo in 2015 [9]. As a direct derivative of photoactivated localization microscopy, ULM possesses the capability to achieve super-resolution imaging based on MB scattering.

## Technical steps of SRUS microvascular imaging

### Microbubble injection

MB possess unique acoustic properties, including high compressibility, mobility, and nonlinear resonance, which distinguish them from tissue scatterers [15] and render them effective as ultrasound contrast agents [9]. The core of MB consists of an inert gas that can be exhaled through the lungs, eliminating the need for renal excretion [6]. They can be safely administered to patients, including pregnant women and children, with an extremely low incidence of adverse events [16]. Cluster injection and drip infusion are two commonly methods for introducing MB into the body. Successful SRUS imaging enquires a careful balance between MB concentration and data acquisition time. Lower concentrations enhance spatial resolution by reducing overlap, thereby aiding localization, but they also prolong imaging time [17]. Conversely, higher concentrations theoretically decrease imaging time but can lead to increased spatial overlap and signal loss due to unsuccessful localizations. Employing sparsity-based and deep learning methods can facilitate the use of higher MB concentrations, ultimately enhancing imaging efficiency [5].

### Image reconstruction

After emitting B-mode ultrasound pulses into a medium containing MBs, streaming images of the MB are acquired at regular or ultra-fast frame rates. Image data are collected based on a matrix or beam formation of RF data for each channel [18]. The RF channel data is subsequently demodulated to produce in-phase and quadrature signals, followed by the application of a delay-and-sum algorithm for beamforming [19]. B-mode images are generated by calculating the absolute values of these signals.

### Motion correction

Motion correction is essential for preserving image quality and ensuring precise MB localization as imaging can be adversely affected by factors such as breathing, heartbeat, and tissue motion. Currently, the most prevalent methods for motion correction include Kalman filtering [20], deconvolution [21], phase correlation algorithms [22, 23], spatial–temporal clutter filtering techniques [24], and Doppler-based motion estimation techniques [25]. However, in animal experiments, anesthesia is frequently employed to immobilize the subjects. The choice and dosage of the anesthetic can influence physiological parameters m potentially distorting the measurements of ULM, such as artery diameter, density, and flow velocity, particularly in the brain [26]. The venous supply is more significantly impacted by anesthesia than the arterial supply, with the midbrain exhibiting the most pronounced effects [26]. To account for the movement of animals in an awake state, which better reflects their typical physiological condition, Wang et al. utilized an algorithm known as Open 3-demensional (3D) ULM [27].

### Microbubble detection

Separating MB from the surrounding tissue is a crucial step that ensures accurate mapping of the final image by creating suitable areas for more precise localization. Excessive error signals are a primary source of image noise and can disrupt the subsequent filtering process [18]. The frequency employed, which is influenced by the imaging depth, often dictates the method used to extract MB signals. At higher frequencies, MB exhibit poor resonance properties and scatter harmonics weakly, making techniques based on MB motion or destruction more advantageous. Additionally, separating MB from tissues can be achieved using deep learning approaches and spatiotemporal filtering through singular value decomposition [28].

### Microbubble isolation

Harmonic imaging [29], differential imaging [30], pulse inversion, amplitude modulation [29, 31], and MB signal separation approaches [32] have been employed in early research to differentiate MB, with the last one significantly improve the accuracy and efficiency of detecting MB events within a short acquisition time. However, reducing the concentration of MB in the blood remains the simplest and most effective method for achieving successful signal separation. Continuous injections of MB help maintain a stable concentration, thereby minimizing the likelihood of signal overlap.

### Localization

To obtain accurate coordinates, Gaussian fitting [33], normalized cross-correlation [34], and centroid methods [35] are widely utilized classical localization approaches. Recent advancements have concentrated on improved localization techniques, including sparse reconstruction [36], deep learning [32], sparsity-constrained methods [36], and linear filtering techniques [37, 38], which potentially facilitate higher localization densities.

### Tracking

The tracking procedure involves comparing MB across frames to ascertain the trajectories after establishing their locations in each frame. Various methods, including digital-to-analog tracking techniques [25], spatial–temporal singular value decomposition approaches [36, 39], Hungarian or Kuhn-Munkres algorithms [24], and Kalman filter [40] can be employed for this purpose.

### Visualization

An appropriate interpolation factor must be selected to construct the pixel grid necessary for transforming MB trajectories into super-resolution images. A coarse grid may lead to inaccurate vessel merging and loss of information, while an excessively fine grid can result in blank spaces within the vessels [15]. Lyu et al. proposed the ARU-GAN model, which integrates residual connectivity and attention mechanisms to enhance super-resolution reconstruction tasks [41]. This innovation effectively addresses several challenges in plane-wave ultrasound imaging, including poor image quality, high noise levels, and insufficient contrast.

## Microvascular parameters generated by SRUS

**Fig. 1:**
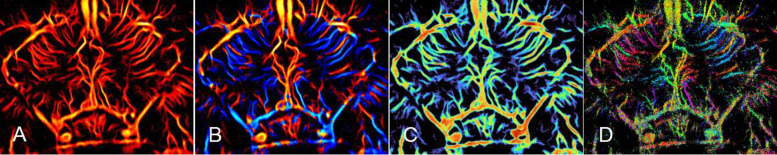
Representative midbrain images of male SD rat generated by super-resolution ultrasound imaging. **A**–**D** represents the vascular density map, vascular orientation map, vascular velocity map, and blood flow angle map, respectively

SRUS achieves approximately a tenfold enhancement in vascular imaging resolution compared to conventional ultrasound imaging [42], while maintaining imaging penetration depth. This advancement effectively circumvents the traditional trade-off between imaging resolution and depth. By tracking the trajectory of MB in blood flow, SRUS can infer both the velocity and direction of micro-blood flow, with a broad spectrum of blood flow velocities, ranging from approximately 1 mm/s to several cm/s [17], allowing for a clearer and more intuitive observation of the microvascular network. However, this capability cannot be achieved by CDFI, SMI, or CEUS. This Furthermore, it mitigates errors caused by traditional Doppler ultrasound, which often arise from angular dependence and spectral spreading [43–45]. This technology can produce detailed maps of microvascular density, blood flow direction, velocity, and microvascular angles, thereby comprehensively revealing the morphology and hemodynamic characteristics of microcirculation (Fig. 1). It quantifies parameters such as vessel density ratio, velocity, complexity, curvature, and perfusion index, providing new insights into the noninvasive acquisition of multidimensional microvascular pathological information and functional assessment of tissue and organs.

## Applications of SRUS imaging

SRUS provides a novel imaging foundation for the indirect assessment of organ and tissue functional status by visualizing microvessel morphology and characterizing structural and functional alterations. Currently, SRUS has been utilized in both animal experiments and clinical research targeting major systemic diseases, and the application/research status of SRUS in various systems is summarized in Table 1.

**Table 1:** Summary of current application of super-resolution ultrasound in animal experiment and clinical application

System	Disease/condition	Subject/model	Author (year)	Country	Target organ	Machine brand	Contrast agent	Means of injection	Parameters on SRUS	Outcome
Neurology	Normal	Rat	Errico et al. 2015 [9]	France	Brain	NA	SonoVue	Injection	Maximum velocity	Quantified the hemodynamics of microvessels (< 10 μm in diameter) in the rodent brain, imaging to a depth of more than 10 mm and displaying vessels with diameters between 15 μ m and 65 μ m
Neurology	Aging	Mouse	Lowerison et al. 2022 [19]	America	Brain	Verasonics Vantage 256	Definity	Injection	Mean velocity, vascular tortuosity, microvascular density	Age has a significant effect on blood flow measurements. Compared with the younger mouse group, the older mouse group had reduced cortical vascular distribution, significantly lower blood flow velocities, and increased tortuosity in several anatomical regions
Neurology	Alzheimer’s disease	Mouse	Lin et al. 2024 [23]	China	Brain	Verasonics Vantage 128HF	Sonovue	Infusion	Vascular density, average velocity of the flow, average vascular length	Vessel density and blood flow velocity were significantly reduced in the posterior medial cortex, visual cortex, and hippocampus, with Aβ accumulation and BBB disruption being most severe in the hippocampus and negatively correlated with vessel density and blood flow velocity
Neurology	Normal	Rat	Renaudin et al. 2022 [46]	France	Brain	Iconeus	Sonovue	Injection	Microbubble count, microbubble flux velocity	Near the cortical surface (< 400 μm), there was a significant increase in blood flow velocity and MB flux, whereas in deeper cortical regions (> 600 μm), there was less change in blood flow velocity but more significant dilation of blood vessel diameter
Neurology	Normal	Rhesus monkeys	Yan et al. 2021 [48]	China	Brain	Verasonics Vantage 256	SonoVue	Injection	Mean blood flow velocity, inner diameters of microvessels	Achieve optimal resolution in the micron range with imaging depths > 35 mm and fast blood velocities of up to 100 cm/s in large intracranial vessels
Neurology	Alzheimer’s disease	Mouse	Lowerison et al. 2024 [50]	America	Brain	Verasonics Vantage 256	Definity	Infusion	Microvascular density, blood flow velocity, vascular tortuosity	Both arterial and venous blood flow velocities were significantly decreased in mice with AD pathology, vascular tortuosity was significantly increased in the hippocampus and internal olfactory cortex, and cerebral vascular dysfunction (decrease in blood flow velocities) preceded the onset of structural changes (decrease in vascular density)
Neurology	Hydrocephalus	Pig	Zhang et al. 2022 [52]	America	Brain	Siemens ACUSON Sequoia	Lumason	Injection	Time-averaged velocity, maximum velocity, number of trajectories	As intracranial pressure increased, cerebral microcirculation decreased significantly in the cortex and thalamus, and more markedly in cortical areas
Digestive system	Cancer	Human	Huang et al. 2021 [20]	America	Pancreas	Mindray Resona 7	SonoVue	Injection	Blood flow velocity and direction vascular density	In a depth of 40–55 mm, the blood supply and drainage vessels of the tumor can be clearly visualized, and vessels with opposite blood flow directions can be distinguished
Digestive system	Normal and acute-on-chronic liver failure	Human	Huang et al. 2021 [20]	America	Liver	Mindray Resona 7	SonoVue	Injection	Blood flow velocity, blood flow direction, vascular density, vascular tortuosity	The vascular structure of the diseased liver is more distorted, with marked tortuosity and thinning of the main vessels, and blood flow velocities ranging from 1 mm/s to 60 mm/s, showing a wide range of hemodynamic information from the large vessels to the microvessels
Digestive system	Deep-seated focal nodular hyperplasia	Human	Zeng et al. 2024 [56]	China	Liver	Mindray Resona 7	SonoVue	Injection	Vascular density, vascular velocity, vsascular direction	Central blood-supplying vascular structures and their branches have higher blood flow velocities and a radial blood flow pattern pointing to the periphery
Digestive system	Cancer	Rat	Brown et al. 2023 [57]	America	Liver	Vevo 3100	Definity	Injection	Microvascular density, tumor size, tumor necrosis rate	With a spatial resolution of up to 34 microns, microvessel density levels and tumor size measurements were significantly lower and smaller, respectively, in complete responders after 14 days compared to partial responders or control animals
Digestive system	Cancer	Rabbit	Zhang et al. 2021 [58]	America	Liver	Verasonics Vantage 256	Definity	Injection	Microvascular density, blood flow velocity, blood flow direction, tissue motion, tumor size	Hypovascularization of the tumor core and hypervascularization of the surrounding area, which continued to grow after sorafenib treatment, with further reduction of vascularization in the core area
Nephrology	Acute kidney injury	Human	Huang et al. 2024 [6]	China	Kidney	Mindray Resona A20	SonoVue	Injection	Microvascular density, serum creatinine	Microvessel density was reduced, the kidney's interlobular artery resistance index rose, and renal cortical microvessel density and serum creatinine significantly correlated negatively
Nephrology	Normal	Human	Huang et al. 2021 [20]	America	Kidney	Mindray Resona 7	SonoVue	Injection	Microvessel density, bi-directional density, velocity magnitude	Clearly distinguishes between adjacent cortical arteries and veins, demonstrating the complex hemodynamic features from cortex to medulla
Nephrology	Normal and kidney transplant	Rats and humans	Denis et al. 2023 [59]	France	Kidney	Verasonics Vantage 256	Sonovue	Infusion	Normalized speed, vascular density	Observation of individual glomerular structures in rat and human kidney transplant recipients revealed that microbubbles in the glomeruli exhibited slower velocity, longer dwell time and lower dispersion
Nephrology	Ischemia	Rat	Andersen et al. 2020 [60]	Denmark	Kidney	BK Medical ApS	SonoVue	Infusion	Flow direction, velocity, vessel diameter	After ischemia–reperfusion, renal microcirculation is compromised, cortical blood flow is decreased, and venous clamping-induced renal microvascular alterations are more noticeable
Nephrology	Acute injury	Mouse	Chen et al. 2020 [61]	America	Kidney	Verasonics Vantage 128	Definity	Injection	Vessel density, microvasculature tortuosity, kidney size, relative blood volume	The kidneys show substantial interstitial fibrosis following renal ischemia–reperfusion injury, with a marked drop in cortical thickness, a marked rise in cortical microvessel tortuosity, and a large decrease in renal volume, relative blood volume, and vascular density
Nephrology	Type 2 diabetes	Rat	Søgaard et al. 2023 [62]	Denmark	Kidney	BK Medical	Sonovue	Infusion	Vascular density, vascular tortuosity	Reduced inner and outer medullary vascular density, increased vascular tortuosity, and cortical vascular density are the hallmarks of early diabetic nephropathy
Nephrology	Hypertensive nephrosclerosis	Rat	Qiu et al. 2022 [63]	China	Kidney	Verasonics Vantage 256	Sonovue	Injection	Blood flow speed	Rats with spontaneous hypertension exhibited narrowing of the lumen, thickening of the tiny arteriolar intima and media, slight atrophy and hyaline degeneration of glomerular capillary collaterals, and noticeably increased total arterial blood flow velocities
Nephrology	Cancer	Chicken	Lowerison et al. 2020 [64]	America	Kidney	Verasonics Vantage 256	SonoVue	Injection	Microvascular density, blood volume, velocity, vascular tortuosity	The chorioallantoic membrane tumor's central portion displays hypoxic characteristics, slower blood flow, and a decreased vascular density
Nephrology	Transplant allograft	Human	Bodard et al. 2023 [65]	France	Kidney	Canon Aplio i800	SonoVue	Injection	Density and directivity maps, blood flow velocity	With a mean minimum detectable vessel diameter of 0.3 ± 0.2 mm, interlobular arteries, arcuate arterioles, cortical radial vessels, and certain medullary structures were observed. As the vessel's distance from the renal envelope increased, the blood flow velocity gradually increased as well
Nephrology	Renal fibrosis	Mouse and human	Hysi et al. 2025 [66]	Canada	Kidney	VevoLAZR-X	NA	Injection	Percentage red pixel density, renal artery blood flow velocity	When evaluating renal fibrosis, H-scan has a high correlation with the histologic gold standard. Compared to the histologic evaluation of local biopsies, which has a poor correlation with post-transplant renal function, it offers a quantitative assessment of whole kidney fibrosis and has a negative correlation with renal function one year after transplantation
Reproductive system	Normal	Female adult sheep	Kanoulas et al. 2019 [68]	America	Ovary	Philips	SonoVue	Injection	Mean velocity, vascular density, vessel diameters	With a resolution enhancement of almost 8.5 times, the smallest blood artery width found was 60 microns
Reproductive system	Pre-menopausal and post-menopausal groups	Human	Wang et al. 2024 [69]	China	Female urethral blood vessels	Mindray Resona R9	SonoVue	Injection	Fractal dimension, vessel proportion, mean velocity, vessel tortuosity index, mean diameter	Postmenopausal women exhibit markedly decreased urethral vascular parameters, including fractal dimension, vascular ratio, mean vessel diameter, and urethral mucosal layer blood flow
Immune system	Normal	Rabbit	Zhu et al. 2019 [70]	England	Lymph node	Verasonics Vantage 256	In-house–manufactured gas-filled microbubbles	Injection	Vessel size, velocity	Most microvessels in rabbit lymph nodes are less than 80 μm in diameter, and most have blood flow velocities of less than 5 mm per second
Immune system	Metastatic cancer	Human	Zhu et al. 2022 [71]	England	Lymph node	Mindray Resona 7S	SonoVue	Injection	Microvessel density,fractal dimension, mean flow speed, local flow direction irregularity	The direction of blood flow was more irregular in metastatic lymph nodes, as evidenced by the slightly greater microvessel density and significantly higher local flow direction irregularity values compared to benign lymph nodes
Cardiovascular system	Heart related disease	Human	Yan et al. 2024 [25]	England	Myocardium	Verasonics	Sonovue	Injection	Vessel diameter, flow-speed distributions	Both long-axis and short-axis images showed distinct myocardial microvascular structures, and intramyocardial blood flow velocities were higher in the middle of major arteries than at their borders, ranging from 27.24 mm/s to 60.46 mm/s
Cardiovascular system	Takayasu arteritis	Human	Goudot et al. 2023 [76]	France	Vasa vasorum	SuperSonic Imagine	Sonovue	Injection	Mean velocity, mean track density, vascular density	The microvascular blood flow velocity varied from 20 to 70 mm/s, with a mean velocity of 40.5 mm/s. those with active takayasu arteritis had considerably more microbubbles and microvessel density within the carotid artery wall than those with quiescent takayasu arteritis
Eyes	Ocular choroidal melanoma	Rat	Quan et al. 2023 [21]	China	Eyes	Verasonics Vantage	Sonovue	Injection	Microvessel density, flow velocity	The microvascular distribution and structure within the ocular choroidal melanoma were clearly visible. The average blood flow velocity is 12.23 mm/s, and the microvascular diameter is smaller than 1 mm
Eyes	Normal and retinal detachment	Rabbit	Lei et al. 2023 [77]	China	Eyes	Verasonics Vantage 256	Sonazoid	Injection	Blood flow velocity, microbubble density, flow direction	Microvessels with diameters as small as 54 μm have been revealed. In the retinas of eyes exhibiting retinal detachment, the retinal layers are disrupted, the arrangement of cells is disordered, the microvascular network is sparser, and abnormal microvascular signals are observed in the vitreous body
Eyes	Elevated intraocular pressure	Rabbit	Qian et al. 2022 [78]	America	Eyes	Verasonics Vantage	Bracco Diagnostics Inc	Injection	Vascular density, mean flow velocity	Under high intraocular pressure, the retinal/choroidal vasculature's vascular density and blood flow velocity are greatly decreased, and tiny blood vessels gradually vanish
Eyes	Elevated intraocular pressure	Rat	Ul Banna et al. 2023 [79]	America	Eyes	FUJIFILM VisualSonics	Trust Bio-sonics	Injection	Perfusion velocity, track density, direction and velocity	Measured vessel diameters were as low as 20 μm. Elevated intraocular pressure significantly decreased arterial flow velocities, with some arteries, such as the central retinal artery, exhibiting a reversal of flow direction, primarily observed during diastole
Superficial organ	Cancer	Human	Huang et al. 2021 [20]	America	Breast	Mindray Resona 7	Sonovue	Injection	Microvessel velocity, microvessel density	Provides morphological and hemodynamic characteristics of breast tumor microvasculature, including information on vessel distribution, orientation, and blood flow velocity
Superficial organ	Thyroid nodules	Human	Zhang et al. 2022 [81]	China	Thyroid	Mindray Resona 9S	Sonovue	Injection	Microvascular flow rate, microvessel density	Benign thyroid nodules have significantly higher microvascular flow rate and microvessel density than malignant thyroid nodules
Superficial organ	Breast lump	Human	Zhang et al. 2022 [82]	China	Breast	Mindray Resona R9	SonoVue	Injection	Microvessel density, microvascular flow rate	The peak intensity, area under the curve, microvessel density, and microvessel flow velocity were significantly higher in malignant breast masses compared to benign masses. Among them, microvessel density exhibited the strongest correlation with the degree of malignancy in breast masses
Superficial organ	Cancer	Human	Opacic et al. 2018 [83]	Germany	Breast	FUJIFILM Visualsonics	SonoVue	Injection	Microvessel density, distribution of vessels, blood flow direction, blood flow velocity	Before chemotherapy, the majority of the tumor's vascularity was located in its edges, with less vascularity in the center. As chemotherapy progressed, the tumor's volume shrank even more, but the degree of vascularization was constant
Skeletal-muscular system	Type 2 diabetes	Mouse	Ghosh et al. 2019 [84]	America	Skeletal muscle	Siemens Acuson Sequoia 512	NA	Infusion	Microvascular density, microvascular blood flow, microbubble count	Insulin-induced microvascular recruitment was markedly enhanced in lean rats during hyperinsulin-normal glucose clamp tests, but this response was markedly reduced in obese rats

### Neurology

To sustain intracellular homeostasis, the metabolic processes of the central nervous system must tightly regulate the release of metabolites and the delivery of nutrients. Neurovascular coupling (NVC) ensures that local blood flow is supplied during neuronal activity to meet energy demands, which is essential for preserving this dynamic equilibrium. Conventional wisdom suggests that the ultrasound does not accurately reflect neuronal activity and function. However, both animal experiments and clinical studies have demonstrated that the emergence of SRUS offers a novel perspective for non-invasive assessment of NVC [46, 47].

#### Age-related changes in the cerebral microcirculation

**Fig. 2:**
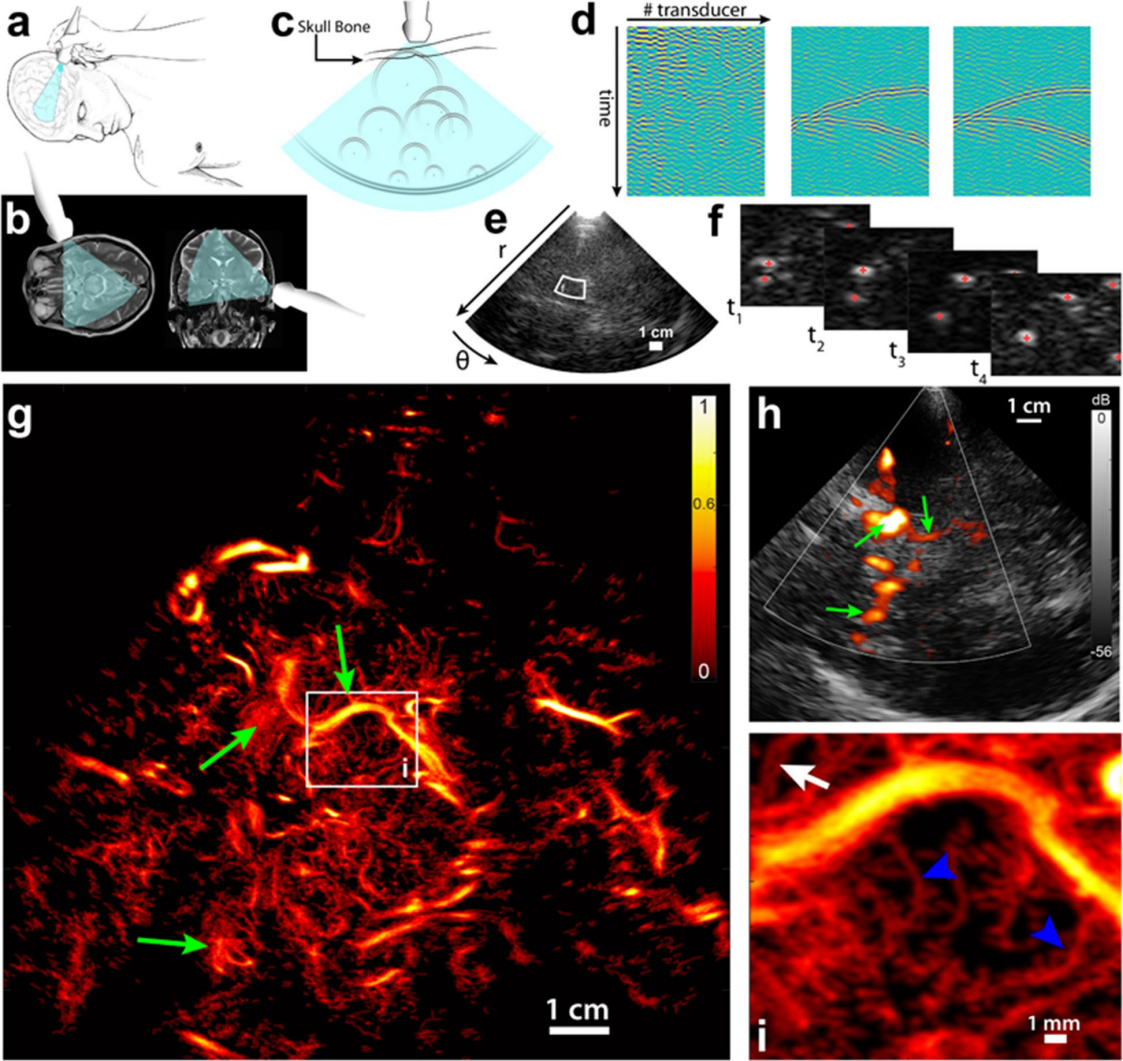
The first application of super-resolution ultrasound imaging in the human brain. Panels **a**–**f** show the steps involved in transducer positioning, field of view establishment, the backscatter of isolated microbubbles as circular waves towards the transducer, aberration correction, image reconstruction, and the localization of individual bubbles, respectively. Panels **g** and **h** display the corresponding images in the density map of ultrasound localization microscopy and the color map of transcranial color-coded sonography, respectively. The green arrows indicate landmarks. Panels **i** presents a zoomed image of panel **g**. (reprineted from Demené et al. [47])

The inaugural intracranial application of SRUS in clinical was pioneered in 2021 by Demené et al., who adeptly harnessed this technology for aneurysm diagnosis (Fig. 2) [47]. Employing a phased array ultrasound probe with a center frequency of 2.93 MHz, SRUS achieved a remarkable imaging depth of up to 120 mm, thereby encompassing the contralateral cerebral cortex. This technique revealed blood flow vortices within the aneurysm as well as the direction and speed of collateral artery blood flow. Compared to traditional transcranial sonography and transcranial color-coded sonography, SRUS can display finer vascular structures while maintaining an equivalent penetration depth. This advancement offers a novel technology for the non-invasive revelation of microcirculation abnormalities.

Lowerison et al. utilized SRUS to demonstrate the physiological changes in cerebral microcirculation associated with aging [19]. In aged mice, a pronounced reduction in global cerebral blood flow velocity was observed, particularly accentuated in the visual cortex and superior colliculus, and vessel tortuosity in the cerebral cortex significantly increased. Yan L et al. reconstructed cerebral microvessels at a resolution of 0.7 mm in monkey [48], and Xing et al. successfully employed 3D ULM to obtain high-resolution transcranial imaging of the macaque brain, achieving a resolution of up to 60.4 μm under craniotomy and dural incision [49]. These two reports represent the application of SRUS in primates, paving the way for clinical application of ULM.

#### Neurodegeneration disease

Alzheimer’s disease (AD) is the most prevalent neurodegenerative disorder, primarily characterized by the deposition of Aβ plaques, and clinical manifestations of dementia. Utilizing the 5xFAD transgenic mouse model, Lowerison et al. [50] and Lin HM et al. [23] investigated the connection between Aβ pathology and the structural and functional alterations of the cerebral vasculature in this AD animal model using ULM. The former study focused more on the temporal relationship between early dysfunction, namely decreased blood flow velocity, and structural changes, specifically decreased vascular density discovering that structural defects in localized vascular density preceded functional decreases in hippocampal and internal olfactory flow velocities. In contrast, the latter study examined the influence of the progression of AD on various brain regions, particularly the posterior medial cortex, both spatially and temporally, as well as the association between vascular changes, Aβ pathology, and disruption of the blood–brain barrier.

#### Assessment of intracranial pressure

The status of cerebral perfusion is crucial for maintaining stable intracranial pressure (ICP), with both parameters being indirectly linked to prognosis and clinical decision-making in various neurosurgical conditions. SRUS facilitates bedside assessment of cerebral microperfusion, enabling early evaluation of neurofunctional recovery and avoiding overly optimistic interpretations derived from conventional angiography or ICP monitoring [51]. By integrating a clinical CEUS system with particle tracking velocimetry, Zhang et al. successfully achieved noninvasive ICP monitoring in hydrocephalic pediatric pig models [52]. In their study, cerebral perfusion was quantified using time-averaged velocity in macro-vessels, as well as a cerebral microcirculation parameter that accounts for the concentration of micro-vessels and their velocity. The results demonstrated a strong correlation between cerebral perfusion and ICP (R^2^ > 0.85). In cases of cerebral ischemia, when ICP exceeds 50% of the mean arterial pressure, the non-dimensionalized cortical microperfusion decreases significantly, by an order of magnitude. This significant reduction in microperfusion can be attributed to the collapse and/or deformation of microvessels due to increased ICP. Such a correlation allows for the inference of ICP levels from changes in cerebral microcirculation parameters [52]. These advancements present a noninvasive method for ICP monitoring, significantly enhancing the early detection and management of ischemic brain injury in clinical practice.

#### Stroke

SRUS has also been utilized in studies addressing stroke, the second leading cause of mortality. Hingot et al. conducted a comparative analysis of monitoring results from 7 T MRI and ultra-fast ultrasound for thrombotic stroke [53]. Their findings indicated that in mouse models, ultra-fast ultrasound imaging, including ultra-fast Doppler and ULM, yielded results comparable to those obtained from MRI. The regions of ischemic damage identified exhibited a high degree of consistency between the two imaging modalities. Furthermore, ultra-fast ultrasound provided more detailed cerebrovascular imaging, effectively delineating the location and extent of hypoperfusion regions. This underscores its potential for non-invasive assessment of post-stroke treatment efficacy.

Another study conducted using mouse model of stroke combined nanodroplet imaging with ULM [54], proposing a novel method for detecting hemorrhagic area. This approach utilizes ultrasound-activated nanodroplets that extravasate and accumulate at the site of hemorrhage to facilitate bleeding detection.

#### Spinal cord imaging

SRUS was also employed to elucidate the characteristics of vascular blood flow changes following spinal cord injury (SCI) in rats at various segments [55]. A total of 9 SD rats were utilized to establish SCI models at different segments using a 50 g aneurysm clip. SRUS revealed that, following SCI, vascular blood flow exhibited distinct changes across different segments of the rats. Specifically, the same injury resulted in the most severe damage to blood vessels in the thoracic spinal cord, followed by the lumbar spinal cord, while the cervical spinal cord exhibited the least damage.

### Digestive system

In the diagnosis and assessment of gastrointestinal disorders, particularly those affecting the liver and pancreas, SRUS has demonstrated considerable promise. Huang et al. investigated the application of SRUS across various human tissues, including healthy livers, livers with acute chronic liver failure, and pancreatic tumors [20]. The SRUS imaging of healthy livers revealed a coherent and systematic vascular network, characterized by smooth transitions between main and branch vessels. In contrast, livers affected by acute and chronic liver damage exhibited distorted and abnormal vascular formations, characterized by constricted branches and even main vessels, resulting in a disordered vascular pattern. These alterations may be closely linked to the pathophysiological mechanisms underlying liver failure.

**Fig. 3:**
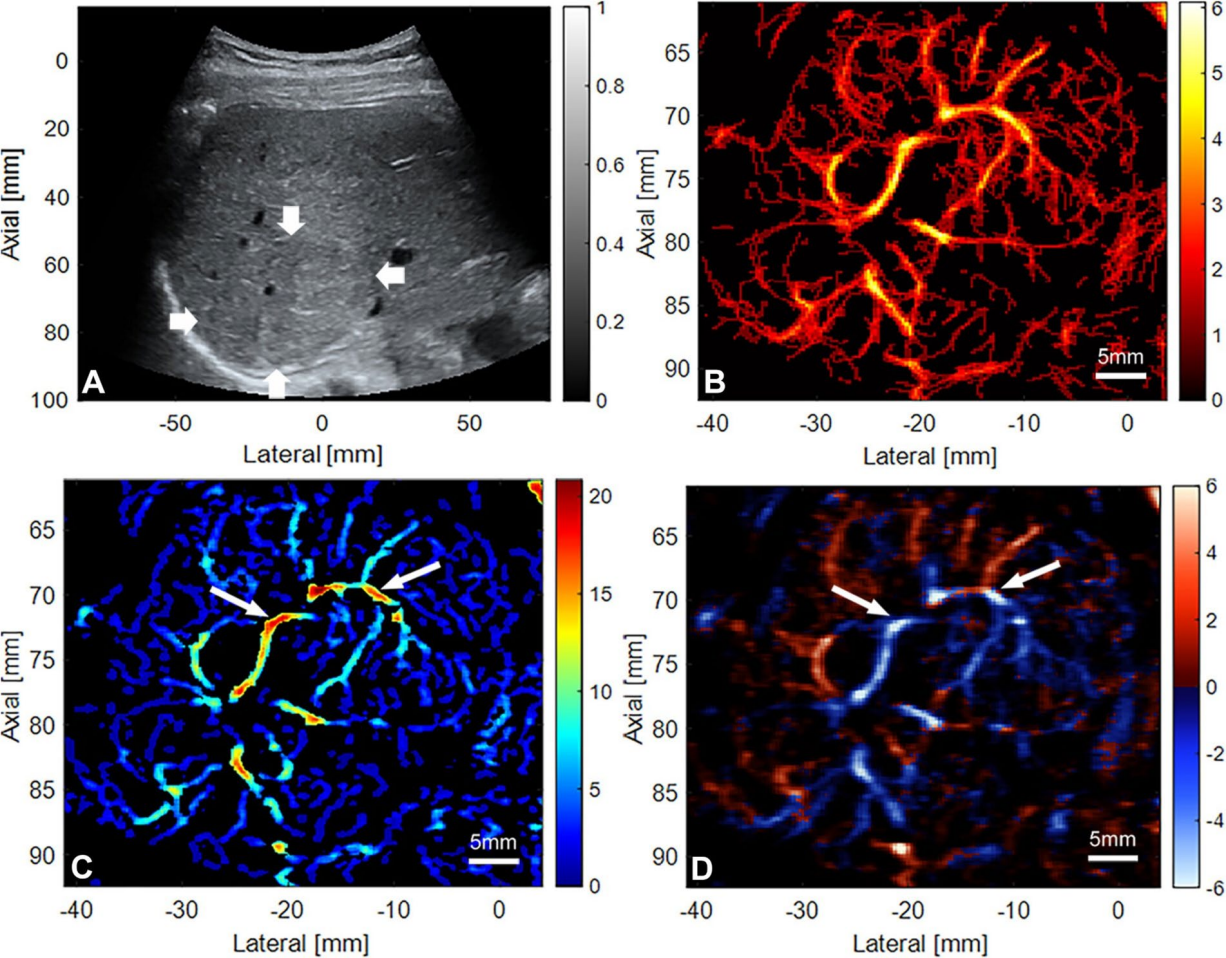
The super-resolution ultrasound imaging of a focal nodular hyperplasia case in a 38-year-old woman. **A**, B-mode ultrasonic image of the mass; **B**, the density map shows abundant microvessels within the nodule; **C**, the blood flow velocity map indicates that the velocity at the center of the nodule is higher than that at the periphery (white arrows), with redder colors representing higher flow velocities; **D**, the direction map reveals a radial blood flow pattern with varying direction, red and blue indicate flow toward and away from the transducer, respectively). (Reprineted from Zeng et al. [56])

Given the pivotal role of angiogenesis in tumor progression, SRUS has emerged as a powerful tool capable of delineating microvascular structures and flow velocities with remarkable precision. This capability positions SRUS a promising method for differentiating between benign and malignant lesions, as well as evaluation treatment efficacy through the analysis of vascular quantity, morphology, distribution, and hemodynamic parameters. The intricate hypervascular distribution and central malformed vascular shunting observed in focal nodular hyperplasia via SRUS correspond with established pathological features (Fig. 3) [56]. Notably, pancreatic tumors imaged at depths of up to 60 mm exhibit increased microvascular density, with altered vascular structure and orientations potentially indicative of tumor growth and aggressiveness [20]. Furthermore, the presence of distorted and thinned vascular structures results in a significant reduction in blood flow velocity within certain tumor vessels, adversely impacting the tumor’s blood supply. In the context of treatment response, Brown et al. noted that, 14 days post-surgery, complete responders in a hepatocellular rat model displayed significantly lower microvascular density and smaller tumor sizes compared to partial responders or controls [57]. Additionally, Zhang et al. demonstrated that, despite continued tumor expansion accompanied by high blood flow velocities and a radial pattern at the periphery, there was a significant decrease in blood flow velocity and vascularization within the tumor core following sorafenib treatment in anti-angiogenic therapy. This was characterized by fewer microvascular events and no notable changes in blood flow direction [58]. The application of SRUS in the liver and pancreas presents a new opportunity for advancing SRUS in conjunction with gastrointestinal endoscopy, thereby facilitating the diagnosis and treatment of non-invasive gastrointestinal diseases in the future.

### Nephrology

The impairment of renal microvascular perfusion is a significant factor in the progression of chronic kidney disease (CKD) and renal fibrosis. Consequently, the detailed microvascular network described by ULM imaging, along with the quantitative microvascular perfusion parameters it provides, particularly for the ability to observe MB flow pattern in the glomeruli in living humans and rats [59], may be more beneficial for assessing chronic kidney diseases compared to CEUS. Andersen et al. demonstrated the utility of SRUS in renal ischemia/reperfusion experiments conducted in rats [60]. After 60 min of reperfusion, SRUS revealed varying degrees of microvascular perfusion recovery. In mice subjected to unilateral ischemia–reperfusion injury [61], kidneys affected by ischemia–reperfusion injury exhibited increased vascular thinning, reduced kidney size, decreased vessel density, diminished blood volume, and heightened cortical vessel tortuosity over time. In human experiments [6], significantly reduced cortical microvascular density was detected by SRUS in the acute kidney injury group, showing a negative correlation with serum creatinine levels.

Diabetes mellitus and hypertension are the primary causes of CKD. Søgaard et al. found that Zucker diabetic fatty rat exhibited a significant reduction in renal cortical vessel density, which was accompanied by a notable increase in proteinuria levels; however, the mean vascular tortuosity index did not differ significantly among the groups [62]. In the context of hypertensive nephrosclerosis, Qiu et al. identified significant higher arteriolar mean blood flow velocities in spontaneously hypertensive rats compared to the Wistar-Kyoto rats [63]. Furthermore, the ability of SRUS to visualize microvascular has been applied in renal cell carcinoma model to find an alternative imaging biomarker for intra-tumor hypoxia [64] and in human renal transplant patients to better assess immune rejection [65]. Using the renal H-scan technique, Hysi et al.'s initial evaluation of transplanted renal fibrosis in humans allows for quick, precise, and noninvasive measurement of the burden of whole kidney fibrosis in both people and mice [66]. The utilization of 3D ULM in rat kidneys has allowed precise volumetric reconstruction of microvascular networks, enabling a comprehensive examination of MB dynamics within both the vasa recta of the medulla and the nephrons [67].

### Reproductive system

The use of SRUS in the reproductive system presents a groundbreaking monitoring tool for forthcoming treatments of senile vaginitis and premature ovarian failure, as well as for elucidating the relationship between micro-perfusion and ovarian and vaginal function. In an experiment conducted by Kanoulas et al. on sheep ovaries, SRUS effectively evaluated the richness and distribution of blood flow by detecting blood vessels as small as 60 µm, thereby clearly visualizing the microvascular architecture within the ovaries [68]. Furthermore, Wang et al. compared the urethral vascular characteristics features of pre-menopausal and post-menopausal women using SRUS [69]. Several urethral vascular metrics, including fractal dimension, vascular ratio, average vascular diameter, maximum blood flow velocity, average blood flow velocity, and vascular tortuosity index were extracted from high-resolution images. Compared to fertile women, the first three parameters were significantly reduced in postmenopausal women. Additionally, these metrics exhibited a trend of increasing with age, followed by a decline. This research provided vital evidence for a deeper understanding of the pathophysiological causes of female urinary incontinence by emphasizing the significant role of urethral blood vessels in female urinary control and examining their changes post-menopause. Nonetheless, infertility may also arise from dysfunctions in male reproductive organs; hence, further research utilizing SRUS in individuals with oligospermia, poor sperm quality, and sexual dysfunction is warranted.

### Immune system

As one of the major immune organs, changes in the microcirculation of lymph nodes may serve as predictors of metastasis. For the first time, Zhu et al. employed 3D SRUS on rabbit lymph nodes in 2019, enabling noninvasive, high-resolution imaging of the microvessels within these nodes [70]. Subsequently, in 2022, the team conducted SRUS on four patients with benign lymph nodes and six patients with metastatic lymph nodes [71]. Their findings indicated that, compared to metastatic lymph nodes, benign lymph nodes exhibited slightly higher mean micro-blood flow velocities, along with lower mean microvessel density and localized flow directional irregularities.

### Cardiovascular system

Historically, myocardial perfusion has been evaluated through coronary angiography, a procedure that involves radiation exposure. In contrast, myocardial ULM may non-invasively enhance our understanding of myocardial microcirculation. Yan et al. were pioneers in employing for both in vitro and in vivo cardiac ULM imaging, conducting studies on two porcine hearts and four human patients [28]. Their findings demonstrated a high level of consistency between the results obtained from SRUS and those from computed tomography angiography. However, in human experiments, participants were instructed to hold their breath to minimize the effects of respiratory motion on cardiac movement, resulting in the collection of only 10 s of data. In the resulting images, certain myocardial regions exhibited an absence of microcirculatory blood flow signals. Further repetitions and external experiments are necessary to elucidate the reasons for the lack of micro-blood flow signals in these regions.

Neovascularization plays a crucial role in the vulnerability of carotid plaques, which are major causes of ischemic stroke [72]. In a study utilizing a rabbit model of atherosclerosis, SRUS quantified intra-plaque trophic vascularization through metrics based on blood flow density ratios [73]. Compared to traditional methods that rely on subjective visual assessments by the human eye to evaluate plaque enhancement in CEUS, SRUS is anticipated to mitigate the limitations associated with inconsistent grading criteria, evolving into a new approach for assessing intra-plaque neovascularization with reduced operator dependence and enhanced standardization [74, 75]. Additionally, Goudot et al. conducted SRUS in 5 patients with active Takayasu’s arteritis (TA) and 11 patients with quiescent TA [76]. They observed visualization of microvessels within the thickened carotid wall, which correlated with the activity of TA.

These approaches may contribute to future aggressive prevention and treatment of ischemic stroke and myocardial infarction by targeting diseased microvessels. The integration of ULM with intravascular ultrasound is anticipated to yield novel diagnostic and therapeutic tools for cardiovascular diseases, particularly concerning coronary atherosclerosis, which is currently assessed in a limited capacity by conventional cardiac ultrasound.

### Eyes

In the visual system, SRUS is invaluable for assessing blood flow velocity and intraocular microvessels as small as 54 µm, which is essential for the early identification of retinal and optic nerve disorders [77]. Through the monitoring of intraocular blood flow, both Qian et al. [78] and UI Banna et al. [79] observed that as intraocular pressure increased, vessel density and flow velocity decreased. The high resolution of SRUS is comparable with optical coherence tomography angiography. Furthermore, SRUS technology allows for high-resolution observation of the microvascular structure and blood flow velocity throughout the entire eye, facilitating the early detection of ocular choroidal melanoma [21].

### Superficial organ

**Fig. 4:**
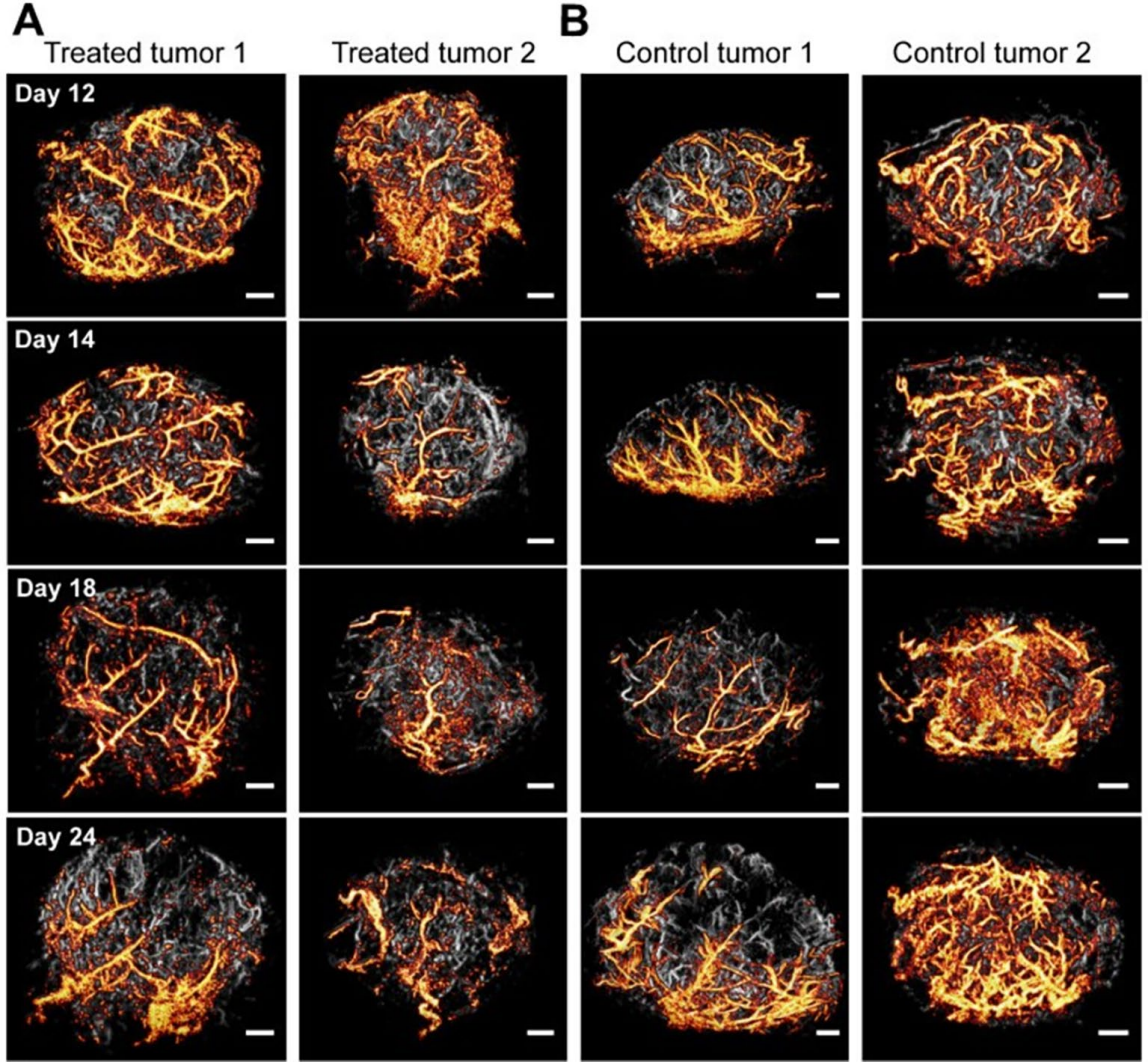
3-dimensional ultrasound localization microscopy revealed morphological changes in microcirculation within a glioblastoma cancer model before and after anti-angiogenic treatment (**A** treatment group; **B** control group). Notably, just 1 day after the initial injection of bevacizumab, a pronounced vascular normalization effect was observed in the treatment group. In contrast, micro-vessel density continued to increase in the control group. (Reprineted from Yin et al. [80])

The application of SRUS in superficial organs primarily leverages microvascular data to differentiate between benign and malignant and to evaluate the therapeutic efficacy post-treatment (Fig. 4) [80]. Research indicates that benign thyroid nodules exhibit significantly higher microvascular flow rates and microvessel densities compared to malignant ones [81]. This distinction arises primarily from the typically adequate blood supply feature of malignant thyroid nodules. In contrast, while malignant breast tumors achieve peak enhancement more rapidly than benign tumors, they also display significant higher microvascular flow rates and microvessel densities [20, 82]. However, these studies did not compare the diagnostic performance of various ultrasound modalities in the differential diagnosis of thyroid/breast masses.

Further study by Opacic et al. have indicated that ULM technology can be employed to monitor post-treatment outcomes [83]. In case of triple negative breast carcinoma, vascularization was predominantly observed in the peripheral regions of the tumor, with only moderate vascular presence in the core and no apparent directional flow prior to treatment. After three cycles of neoadjuvant chemotherapy, a reduction in tumor volume was accompanied by a sustained improvement in vascularization levels.

### Skeletal-muscular system

Through the application of SRUS, Ghosh et al. demonstrated distinct patterns of skeletal muscle microvascular responses to insulin stimulation by comparing the microvessel enlargement of and blood flow augmentation between lean and obese mice models [84]. The study revealed significant microvessel recruitment in the skeletal muscles of young, lean mice following insulin administration, indicating that insulin effectively induces microvascular dilation and enhances perfusion in this group, thereby improving muscular glucose uptake. Conversely, aged obese mice exhibited diminished microvessel recruitment in response to insulin, suggesting a potential connection between obesity-associated insulin resistance, and impaired insulin-mediated microvascular dilation. Compared to the parameters derived from time-intensity curve generated by CEUS, the coefficients of variation of the time MB count curve-derived parameters from SRUS were considerably lower. This discrepancy may stem from difference in the post-processing procedures between SRUS and CEUS.

## Limitations and prospects of SRUS

As an emerging imaging technique, SRUS imaging is undergoing continuous optimization. However, this novel technology still has several limitations. Firstly, acquiring high-frame-rate image information during the imaging process is crucial for capturing the motion trajectory of each MB, which leads to prolonged reconstruction times and increased memory usage. Although deep learning shows considerable promise in handling data, challenges remain, such as the scarcity of training data requiring a ground truth, and discrepancies between training data and actual data [85]. Secondly, the challenge of determining the optimal timing for image acquisition and reconstruction to accurately reflect of the physiological state of organ tissues remains unresolved. This issue is particularly critical because MB is expelled during the respiratory process, resulting in a gradual decrease in their concentration within the tissues. Thirdly, compared to CDFI and CEUS, the imaging process of ULM is primarily not real-time, thus limiting the dynamic observation of microcirculation. The interpretability of ULM parameters requires further elucidation, such as validation through pathological or other imaging methods. Furthermore, in the application of SRUS within the central nervous system, the acoustic beam faces challenges such as attenuation, phase aberration, and propagation difficulties during transmission. The disparity in sound velocity and tissue density between the skull and brain, along with the complex heterogeneous structure of the human skull, contribute to significant distortion of the MB signal, resulting in considerable localization uncertainty. Research into flexible metamaterials has addressed the compatibility issues associated with traditional rigid materials, significantly improving the transcranial transmission rate of acoustic waves [86]. This advancement lays a crucial experimental foundation for the application of SRUS in the neurological field. Looking ahead, the combination of SRUS with functional ultrasound and brain-computer interfaces may unveil new avenues for neuroscience research. Moreover, the integration of SRUS with nanodroplets may offer innovative approaches to tumor treatment and efficacy monitoring.

## Conclusion

In the past decade, SRUS technology has successfully surpassed the diffraction barrier of ultrasound imaging, overcoming the limitations associated with traditional resolution. This advancement has enabled high-precision imaging of micro-blood flow and comprehensive assessment and monitoring of its structural and functional alterations, thereby providing a robust means for elucidating the mechanisms underlying disease generation and development. Although the imaging process and practical applications of SRUS still encounter numerous challenges, current research suggests that SRUS demonstrates significant potential in disease diagnosis, assessment, treatment, and efficacy monitoring.

## Data Availability

Not applicable.

## Abbreviations

AD: Alzheimer’s disease
CDFI: Color Doppler flow imaging
CEUS: Contrast-enhanced ultrasound
CKD: Chronic kidney disease
ICP: Intracranial pressure
MB: Microbubble
NVC: Neurovascular coupling
OCM: Ocular choroidal melanoma
PSF: Point spread function
RF: Radio frequency
RA: Rheumatoid arthritis
SCI: Spinal cord injury
SRUS: Super-resolution ultrasound
TA: Takayasu’s arteritis
3D: 3-Dimensional

## References

[1] Rasmussen LD, Westra J, Karim SR et al (2025) Microvascular resistance reserve: impact on health status and myocardial perfusion after revascularization in chronic coronary syndrome. Eur Heart J 46(5):424–435. 10.1093/eurheartj/ehae604 39217607 10.1093/eurheartj/ehae604

[2] Sablik M, Sannier A, Raynaud M et al (2025) Microvascular inflammation of kidney allografts and clinical outcomes. N Engl J Med 392(8):763–776. 10.1056/NEJMoa2408835 39450752 10.1056/NEJMoa2408835

[3] Souza A, Troschel AS, Marquardt JP et al (2025) Skeletal muscle adiposity, coronary microvascular dysfunction, and adverse cardiovascular outcomes. Eur Heart J 46(12):1112–1123. 10.1093/eurheartj/ehae827 39827905 10.1093/eurheartj/ehae827 PMC13376131

[4] Tang MX, Mulvana H, Gauthier T et al (2011) Quantitative contrast-enhanced ultrasound imaging: a review of sources of variability. Interface Focus 1(4):520–539. 10.1098/rsfs.2011.0026 22866229 10.1098/rsfs.2011.0026 PMC3262271

[5] Xia S, Zheng Y, Hua Q et al (2024) Super-resolution ultrasound and microvasculomics: a consensus statement. Eur Radiol 34(11):7503–7513. 10.1007/s00330-024-10796-3 38811389 10.1007/s00330-024-10796-3

[6] Huang X, Zhang Y, Zhou Q, Deng Q (2024) Value of ultrasound super-resolution imaging for the assessment of renal microcirculation in patients with acute kidney injury: a preliminary study. Diagnostics (Basel) 14(11):1192. 10.3390/diagnostics14111192 38893718 10.3390/diagnostics14111192 PMC11171740

[7] Xia S, Hua Q, Song Y et al (2025) Super-resolution ultrasound imaging of intranodal lymphatic sinuses for predicting sentinel lymph node metastasis in breast cancer: a preliminary study. Eur Radiol. 10.1007/s00330-025-11520-5 40186632 10.1007/s00330-025-11520-5

[8] Ikeda O, Sato T, Suzuki K (1979) Super-resolution imaging system using waves with a limited frequency bandwidth. J Acoust Soc Am 65:75–81. 10.1121/1.382270

[9] Errico C, Pierre J, Pezet S et al (2015) Ultrafast ultrasound localization microscopy for deep super-resolution vascular imaging. Nature 527(7579):499–502. 10.1038/nature16066 26607546 10.1038/nature16066

[10] Betzig E, Patterson GH, Sougrat R et al (2006) Imaging intracellular fluorescent proteins at nanometer resolution. Science 313(5793):1642–1645. 10.1126/science.1127344 16902090 10.1126/science.1127344

[11] Hess ST, Girirajan TP, Mason MD (2006) Ultra-high resolution imaging by fluorescence photoactivation localization microscopy. Biophys J 91(11):4258–4272. 10.1529/biophysj.106.091116 16980368 10.1529/biophysj.106.091116 PMC1635685

[12] Rust MJ, Bates M, Zhuang X (2006) Sub-diffraction-limit imaging by stochastic optical reconstruction microscopy (STORM). Nat Methods 3(10):793–795. 10.1038/nmeth929 16896339 10.1038/nmeth929 PMC2700296

[13] Zheng Y, Krupka T, Wu H, Wang Z, Exner AA (2009) 0079: Direct measurement of blood flow velocity in small diameter vessels using contrast-enhanced ultrasound. Ultrasound Med Biol 35(8):S16. 10.1016/j.ultrasmedbio.2009.06.063

[14] Couture O, Besson B, Montaldo G, Fink M & Tanter M. In 2011 IEEE International Ultrasonics Symposium. 1285–1287.

[15] Lowerison M, Shin Y, Song P (2024) Super-resolution ultrasound imaging: the quest for microvessels. Acoustics Today 20(3):20. 10.1121/AT.2024.20.3.20

[16] Shang Y, Xie X, Luo Y et al (2023) Safety findings after intravenous administration of sulfur hexafluoride microbubbles to 463,434 examinations at 24 centers. Eur Radiol 33(2):988–995. 10.1007/s00330-022-09108-4 36205769 10.1007/s00330-022-09108-4

[17] Yi HM, Lowerison MR, Song PF, Zhang W (2022) A review of clinical applications for super-resolution ultrasound localization microscopy. Curr Med Sci 42(1):1–16. 10.1007/s11596-021-2459-2 35167000 10.1007/s11596-021-2459-2

[18] Shekhawat GS, Dravid VP (2005) Nanoscale imaging of buried structures via scanning near-field ultrasound holography. Science 310(5745):89–92. 10.1126/science.1117694 16210534 10.1126/science.1117694

[19] Lowerison MR, Sekaran NVC, Zhang W et al (2022) Aging-related cerebral microvascular changes visualized using ultrasound localization microscopy in the living mouse. Sci Rep 12(1):619. 10.1038/s41598-021-04712-8 35022482 10.1038/s41598-021-04712-8 PMC8755738

[20] Huang C, Zhang W, Gong P et al (2021) Super-resolution ultrasound localization microscopy based on a high frame-rate clinical ultrasound scanner: an in-human feasibility study. Phys Med Biol. 10.1088/1361-6560/abef45 33725687 10.1088/1361-6560/abef45 PMC8486312

[21] Quan B, Liu X, Zhao S et al (2023) Detecting early ocular choroidal melanoma using ultrasound localization microscopy. Bioengineering (Basel) 10(4):428. 10.3390/bioengineering10040428 37106615 10.3390/bioengineering10040428 PMC10136200

[22] Hingot V, Errico C, Tanter M, Couture O (2017) Subwavelength motion-correction for ultrafast ultrasound localization microscopy. Ultrasonics 77:17–21. 10.1016/j.ultras.2017.01.008 28167316 10.1016/j.ultras.2017.01.008

[23] Lin H, Wang Z, Liao Y et al (2024) Super-resolution ultrasound imaging reveals temporal cerebrovascular changes with disease progression in female 5×FAD mouse model of Alzheimer’s disease: correlation with pathological impairments. EBioMedicine 108:105355. 10.1016/j.ebiom.2024.105355 39293213 10.1016/j.ebiom.2024.105355 PMC11424966

[24] Zheng H, Niu L, Qiu W et al (2023) The emergence of functional ultrasound for noninvasive brain-computer interface. Research 6:0200. 10.34133/research.0200 37588619 10.34133/research.0200 PMC10427153

[25] Yan J, Huang B, Tonko J et al (2024) Transthoracic ultrasound localization microscopy of myocardial vasculature in patients. Nat Biomed Eng 8(6):689–700. 10.1038/s41551-024-01206-6 38710839 10.1038/s41551-024-01206-6 PMC11250254

[26] Wang Y, Lowerison MR, Huang Z et al (2024) Longitudinal awake imaging of mouse deep brain microvasculature with super-resolution ultrasound localization microscopy. bioRxiv. 10.1101/2023.09.01.55578940093181

[27] Chabouh G, Denis L, Abioui-Mourgues M et al. An open-source platform for 3D transcranial Ultrasound Localization Microscopy in awake mice. 2024. 10.1038/s44172-025-00415-4 PMC12144258 40481246

[28] Favre H, Pernot M, Tanter M, Papadacci C (2023) Transcranial 3D ultrasound localization microscopy using a large element matrix array with a multi-lens diffracting layer: anin vitrostudy. Phys Med Biol. 10.1088/1361-6560/acbde3 36808924 10.1088/1361-6560/acbde3

[29] Viessmann OM, Eckersley RJ, Christensen-Jeffries K, Tang MX, Dunsby C (2013) Acoustic super-resolution with ultrasound and microbubbles. Phys Med Biol 58(18):6447. 10.1088/0031-9155/58/18/6447 23999099 10.1088/0031-9155/58/18/6447

[30] Desailly Y, Couture O, Fink M, Tanter M (2013) Sono-activated ultrasound localization microscopy. Appl Phys Lett. 10.1063/1.4826597

[31] Simpson DH, Chin CT, Burns PN (1999) Pulse inversion doppler: a new method for detecting nonlinear echoes from microbubble contrast agents. IEEE Trans Ultrason Ferroelectr Freq Control 46(2):372–382. 10.1109/58.753026 18238434 10.1109/58.753026

[32] Huang C, Lowerison MR, Trzasko JD et al (2020) Short acquisition time super-resolution ultrasound microvessel imaging via microbubble separation. Sci Rep 10(1):6007. 10.1038/s41598-020-62898-9 32265457 10.1038/s41598-020-62898-9 PMC7138805

[33] Heiles B, Chavignon A, Hingot V et al (2022) Performance benchmarking of microbubble-localization algorithms for ultrasound localization microscopy. Nat Biomed Eng 6(5):605–616. 10.1038/s41551-021-00824-8 35177778 10.1038/s41551-021-00824-8

[34] Song P, Trzasko JD, Manduca A et al (2018) Improved super-resolution ultrasound microvessel imaging with spatiotemporal nonlocal means filtering and bipartite graph-based microbubble tracking. IEEE Trans Ultrason Ferroelectr Freq Control 65(2):149–167. 10.1109/tuffc.2017.2778941 29389649 10.1109/TUFFC.2017.2778941 PMC5798010

[35] Christensen-Jeffries K, Browning RJ, Tang MX, Dunsby C, Eckersley RJ (2015) In vivo acoustic super-resolution and super-resolved velocity mapping using microbubbles. IEEE Trans Med Imaging 34(2):433–440. 10.1109/tmi.2014.2359650 25265604 10.1109/TMI.2014.2359650

[36] Zhao S, Hartanto J, Joseph R et al (2023) Hybrid photoacoustic and fast super-resolution ultrasound imaging. Nat Commun 14(1):2191. 10.1038/s41467-023-37680-w 37072402 10.1038/s41467-023-37680-w PMC10113238

[37] Bar-Zion A, Solomon O, Tremblay-Darveau C, Adam D, Eldar YC (2018) SUSHI: sparsity-based ultrasound super-resolution hemodynamic imaging. IEEE Trans Ultrason Ferroelectr Freq Control 65(12):2365–2380. 10.1109/tuffc.2018.2873380 30295619 10.1109/TUFFC.2018.2873380

[38] Harput S, Christensen-Jeffries K, Brown J et al. In 2017 IEEE International Ultrasonics Symposium (IUS). 1–4. 10.1109/ULTSYM.2018.8580145 PMC7610905 34093969

[39] Gao F, Li B, Chen L et al (2024) Ultrasound image super-resolution reconstruction based on semi-supervised CycleGAN. Ultrasonics 137:107177. 10.1016/j.ultras.2023.107177 37832382 10.1016/j.ultras.2023.107177

[40] Liu J, Liang M, Ma J et al (2025) Microbubble tracking based on partial smoothing-based adaptive generalized labelled Multi-Bernoulli filter for super-resolution imaging. Ultrasonics 145:107455. 10.1016/j.ultras.2024.107455 39332248 10.1016/j.ultras.2024.107455

[41] Lyu Y, Jiang X, Xu Y et al (2023) ARU-GAN: U-shaped GAN based on attention and residual connection for super-resolution reconstruction. Comput Biol Med 164:107316. 10.1016/j.compbiomed.2023.107316 37595521 10.1016/j.compbiomed.2023.107316

[42] Desailly Y, Pierre J, Couture O, Tanter M (2015) Resolution limits of ultrafast ultrasound localization microscopy. Phys Med Biol 60(22):8723–8740. 10.1088/0031-9155/60/22/8723 26509596 10.1088/0031-9155/60/22/8723

[43] Hoyt K, Sorace A, Saini R (2012) Quantitative mapping of tumor vascularity using volumetric contrast-enhanced ultrasound. Invest Radiol 47(3):167–174. 10.1097/RLI.0b013e318234e6bc 22104962 10.1097/RLI.0b013e318234e6bc PMC3288214

[44] Huang SF, Chang RF, Moon WK et al (2008) Analysis of tumor vascularity using three-dimensional power doppler ultrasound images. IEEE Trans Med Imaging 27(3):320–330. 10.1109/TMI.2007.904665 18334428 10.1109/TMI.2007.904665

[45] Mahoney M, Sorace A, Warram J, Samuel S, Hoyt K (2014) Volumetric contrast-enhanced ultrasound imaging of renal perfusion. J Ultrasound Med 33(8):1427–1437. 10.7863/ultra.33.8.1427 25063408 10.7863/ultra.33.8.1427 PMC4135386

[46] Renaudin N, Demene C, Dizeux A et al (2022) Functional ultrasound localization microscopy reveals brain-wide neurovascular activity on a microscopic scale. Nat Methods 19(8):1004–1012. 10.1038/s41592-022-01549-5 35927475 10.1038/s41592-022-01549-5 PMC9352591

[47] Demene C, Robin J, Dizeux A et al (2021) Transcranial ultrafast ultrasound localization microscopy of brain vasculature in patients. Nat Biomed Eng 5(3):219–228. 10.1038/s41551-021-00697-x 33723412 10.1038/s41551-021-00697-x PMC7610356

[48] Yan L, Bai C, Zheng Y et al (2021) Study on the application of super-resolution ultrasound for cerebral vessel imaging in rhesus monkeys. Front Neurol 12:720320. 10.3389/fneur.2021.720320 34867712 10.3389/fneur.2021.720320 PMC8637903

[49] Xing P, Perrot V, Dominguez-Vargas AU et al (2025) 3D ultrasound localization microscopy of the nonhuman primate brain. EBioMedicine 111:105457. 10.1016/j.ebiom.2024.105457 39708427 10.1016/j.ebiom.2024.105457 PMC11730257

[50] Lowerison MR, Vaithiyalingam Chandra Sekaran N, Dong Z et al (2024) Super-resolution ultrasound reveals cerebrovascular impairment in a mouse model of Alzheimer’s disease. J Neurosci 44(9):e1251232024. 10.1523/jneurosci.1251-23.2024 38253533 10.1523/JNEUROSCI.1251-23.2024 PMC10904092

[51] Huang W, Hua C, Guo Y et al (2023) Super resolution imaging reconstruction reveals that gold standard methods may not correctly conclude neural/brain functional recovery. Comput Med Imaging Graph 105:102198. 10.1016/j.compmedimag.2023.102198 36805708 10.1016/j.compmedimag.2023.102198

[52] Zhang Z, Hwang M, Kilbaugh TJ, Sridharan A, Katz J (2022) Cerebral microcirculation mapped by echo particle tracking velocimetry quantifies the intracranial pressure and detects ischemia. Nat Commun 13(1):666. 10.1038/s41467-022-28298-5 35115552 10.1038/s41467-022-28298-5 PMC8814032

[53] Hingot V, Brodin C, Lebrun F et al (2020) Early ultrafast ultrasound imaging of cerebral perfusion correlates with ischemic stroke outcomes and responses to treatment in mice. Theranostics 10(17):7480–7491. 10.7150/thno.44233 32685000 10.7150/thno.44233 PMC7359089

[54] Lin BZ, Fan AC, Wang Y et al (2025) Combined nanodrops imaging and ultrasound localization microscopy for detecting intracerebral hemorrhage. Ultrasound Med Biol 51(4):707–714. 10.1016/j.ultrasmedbio.2025.01.002 39837748 10.1016/j.ultrasmedbio.2025.01.002 PMC12615983

[55] Dong HR, Yu JJ, Chen XY, Xu KL, Xie R (2024) Application of super-resolution and ultrafast ultrasound to reveal the characteristics of vascular blood flow changes after rat spinal cord injury at different segments. Zhonghua Yi Xue Za Zhi 104(9):690–694. 10.3760/cma.j.cn112137-20231020-00830 38418168 10.3760/cma.j.cn112137-20231020-00830

[56] Zeng QQ, Liang P (2024) Super-resolution US imaging of focal nodular hyperplasia. Radiology 311(1):e233130. 10.1148/radiol.233130 38687219 10.1148/radiol.233130

[57] Brown KG, Li J, Margolis R et al (2023) Assessment of transarterial chemoembolization using super-resolution ultrasound imaging and a rat model of hepatocellular carcinoma. Ultrasound Med Biol 49(5):1318–1326. 10.1016/j.ultrasmedbio.2023.01.021 36868958 10.1016/j.ultrasmedbio.2023.01.021 PMC10257127

[58] Zhang W, Lowerison MR, Dong Z et al (2021) Super-resolution ultrasound localization microscopy on a rabbit liver VX2 tumor model: an initial feasibility study. Ultrasound Med Biol 47(8):2416–2429. 10.1016/j.ultrasmedbio.2021.04.012 34045095 10.1016/j.ultrasmedbio.2021.04.012 PMC8278629

[59] Denis L, Bodard S, Hingot V et al (2023) Sensing ultrasound localization microscopy for the visualization of glomeruli in living rats and humans. EBioMedicine 91:104578. 10.1016/j.ebiom.2023.104578 37086650 10.1016/j.ebiom.2023.104578 PMC10149190

[60] Andersen SB, Taghavi I, Hoyos CAV et al (2020) Super-resolution imaging with ultrasound for visualization of the renal microvasculature in rats before and after renal ischemia: a pilot study. Diagnostics (Basel) 10(11):862. 10.3390/diagnostics10110862 33105888 10.3390/diagnostics10110862 PMC7690607

[61] Chen Q, Yu J, Rush BM et al (2020) Ultrasound super-resolution imaging provides a noninvasive assessment of renal microvasculature changes during mouse acute kidney injury. Kidney Int 98(2):355–365. 10.1016/j.kint.2020.02.011 32600826 10.1016/j.kint.2020.02.011 PMC7387159

[62] Søgaard SB, Andersen SB, Taghavi I et al (2023) Super-resolution ultrasound imaging of renal vascular alterations in zucker diabetic fatty rats during the development of diabetic kidney disease. Diagnostics (Basel) 13(20):3197. 10.3390/diagnostics13203197 37892017 10.3390/diagnostics13203197 PMC10605617

[63] Qiu L, Zhang J, Yang Y et al (2022) In vivo assessment of hypertensive nephrosclerosis using ultrasound localization microscopy. Med Phys 49(4):2295–2308. 10.1002/mp.15583 35218672 10.1002/mp.15583

[64] Lowerison MR, Huang C, Lucien F, Chen S, Song P (2020) Ultrasound localization microscopy of renal tumor xenografts in chicken embryo is correlated to hypoxia. Sci Rep 10(1):2478. 10.1038/s41598-020-59338-z 32051485 10.1038/s41598-020-59338-z PMC7015937

[65] Bodard S, Denis L, Hingot V et al (2023) Ultrasound localization microscopy of the human kidney allograft on a clinical ultrasound scanner. Kidney Int 103(5):930–935. 10.1016/j.kint.2023.01.027 36841476 10.1016/j.kint.2023.01.027

[66] Hysi E, Baek J, Koven A et al (2025) A first-in-human study of quantitative ultrasound to assess transplant kidney fibrosis. Nat Med 31(3):970–978. 10.1038/s41591-024-03417-5 40033112 10.1038/s41591-024-03417-5 PMC11922760

[67] Chabouh G, Denis L, Bodard S et al (2024) Whole organ volumetric sensing ultrasound localization microscopy for characterization of kidney structure. IEEE Trans Med Imaging 43(11):4055–4063. 10.1109/tmi.2024.3411669 38857150 10.1109/TMI.2024.3411669

[68] Kanoulas E, Butler M, Rowley C et al (2019) Super-resolution contrast-enhanced ultrasound methodology for the identification of in vivo vascular dynamics in 2D. Invest Radiol 54(8):500–516. 10.1097/rli.0000000000000565 31058661 10.1097/RLI.0000000000000565 PMC6661242

[69] Wang X, Hua C, Ying T et al (2024) Super-resolution imaging of urethral vasculature in healthy pre- and post-menopausal females. iScience 27(3):109310. 10.1016/j.isci.2024.109310 38482493 10.1016/j.isci.2024.109310 PMC10933541

[70] Zhu J, Rowland EM, Harput S et al (2019) 3D super-resolution US imaging of rabbit lymph node vasculature in vivo by using microbubbles. Radiology 291(3):642–650. 10.1148/radiol.2019182593 30990382 10.1148/radiol.2019182593

[71] Zhu J, Zhang C, Christensen-Jeffries K et al (2022) Super-resolution ultrasound localization microscopy of microvascular structure and flow for distinguishing metastatic lymph nodes - an initial human study. Ultraschall Med 43(6):592–598. 10.1055/a-1917-0016 36206774 10.1055/a-1917-0016

[72] Hou C, M-x Li, He W (2024) Carotid plaque-RADS: a novel stroke risk classification system. JACC Cardiovasc Imaging 17(2):226. 10.1016/j.jcmg.2023.11.007 38325960 10.1016/j.jcmg.2023.11.007

[73] Yu J, Lavery L, Kim K (2018) Super-resolution ultrasound imaging method for microvasculature in vivo with a high temporal accuracy. Sci Rep 8(1):13918. 10.1038/s41598-018-32235-2 30224779 10.1038/s41598-018-32235-2 PMC6141566

[74] Hou C, Xuan JQ, Zhao L et al (2024) Comparison of the diagnostic performance of contrast-enhanced ultrasound and high-resolution magnetic resonance imaging in the evaluation of histologically defined vulnerable carotid plaque: a systematic review and meta-analysis. Quant Imaging Med Surg 14(8):5814–5830. 10.21037/qims-24-540 39143999 10.21037/qims-24-540 PMC11320555

[75] Leroy H, Wang LZ, Jimenez A et al (2025) Assessment of microvascular flow in human atherosclerotic carotid plaques using ultrasound localization microscopy. EBioMedicine 111:105528. 10.1016/j.ebiom.2024.105528 39729884 10.1016/j.ebiom.2024.105528 PMC11733184

[76] Goudot G, Jimenez A, Mohamedi N et al (2023) Assessment of Takayasu’s arteritis activity by ultrasound localization microscopy. EBioMedicine 90:104502. 10.1016/j.ebiom.2023.104502 36893585 10.1016/j.ebiom.2023.104502 PMC10017361

[77] Lei S, Zhang C, Zhu B et al (2023) In vivo ocular microvasculature imaging in rabbits with 3D ultrasound localization microscopy. Ultrasonics 133:107022. 10.1016/j.ultras.2023.107022 37178486 10.1016/j.ultras.2023.107022

[78] Qian X, Huang C, Li R et al (2022) Super-resolution ultrasound localization microscopy for visualization of the ocular blood flow. IEEE Trans Biomed Eng 69(5):1585–1594. 10.1109/tbme.2021.3120368 34652993 10.1109/TBME.2021.3120368 PMC9113921

[79] Ul Banna H, Mitchell B, Chen S, Palko J (2023) Super-resolution ultrasound localization microscopy using high-frequency ultrasound to measure ocular perfusion velocity in the rat eye. Bioengineering (Basel) 10(6):689. 10.3390/bioengineering10060689 37370620 10.3390/bioengineering10060689 PMC10295416

[80] Yin J, Dong F, An J et al (2024) Pattern recognition of microcirculation with super-resolution ultrasound imaging provides markers for early tumor response to anti-angiogenic therapy. Theranostics 14(3):1312–1324. 10.7150/thno.89306 38323316 10.7150/thno.89306 PMC10845201

[81] Zhang G, Yu J, Lei YM et al (2022) Ultrasound super-resolution imaging for the differential diagnosis of thyroid nodules: a pilot study. Front Oncol 12:978164. 10.3389/fonc.2022.978164 36387122 10.3389/fonc.2022.978164 PMC9647016

[82] Zhang G, Lei YM, Li N et al (2022) Ultrasound super-resolution imaging for differential diagnosis of breast masses. Front Oncol 12:1049991. 10.3389/fonc.2022.1049991 36408165 10.3389/fonc.2022.1049991 PMC9669901

[83] Opacic T, Dencks S, Theek B et al (2018) Motion model ultrasound localization microscopy for preclinical and clinical multiparametric tumor characterization. Nat Commun 9(1):1527. 10.1038/s41467-018-03973-8 29670096 10.1038/s41467-018-03973-8 PMC5906644

[84] Ghosh D, Peng J, Brown K et al (2019) Super-resolution ultrasound imaging of skeletal muscle microvascular dysfunction in an animal model of type 2 diabetes. J Ultrasound Med 38(10):2589–2599. 10.1002/jum.14956 30706511 10.1002/jum.14956 PMC6669112

[85] Zhang G, Hu X, Ren X et al (2024) In vivo ultrasound localization microscopy for high-density microbubbles. Ultrasonics 143:107410. 10.1016/j.ultras.2024.107410 39084108 10.1016/j.ultras.2024.107410

[86] Chen J, Liu B, Peng G et al (2025) Achieving high-performance transcranial ultrasound transmission through Mie and Fano resonance in flexible metamaterials. Adv Sci (Weinh). 10.1002/advs.202500170 40135785 10.1002/advs.202500170 PMC12097009

